# 1-{(1*Z*)-1-[6-(4-Chloro­phen­oxy)hex­y­l­oxy]-1-(2,4-difluoro­phen­yl)prop-1-en-2-yl}-1*H*-1,2,4-triazol-4-ium nitrate

**DOI:** 10.1107/S1600536811034933

**Published:** 2011-09-14

**Authors:** Wen-qian Wan, Kai Wang, Yong-hong Hu, Fei Shen, Wen-ge Yang

**Affiliations:** a Jiangsu Engineering Technology Research Center of Polypeptide Pharmaceutical, College of Life Science and Pharmaceutical Engineering, Nanjing University of Technology, Xinmofan Road No. 5 Nanjing, Nanjing 210009, People’s Republic of China

## Abstract

In the title compound, C_23_H_25_ClF_2_N_3_O_2_^+^·NO_3_^−^, the triazole ring makes dihedral angles of 60.9 (4) and 25.0 (3)° with the 6-chloro­phenyl and 2,4-difluoro­phenyl rings, respectively. The mol­ecule adopts a *Z* configuration about the C=C double bond. In the crystal, the cations and anions are linked by N—H⋯O hydrogen bonds and weak C—H⋯O inter­actions.

## Related literature

For the use of triazole derivatives as anti­fungal agents, see: Jeu *et al.* (2003 ▶); Fromtling & Castaner (1996 ▶). For the synthesis, see: Zirngibl & Thiele (1985 ▶). 
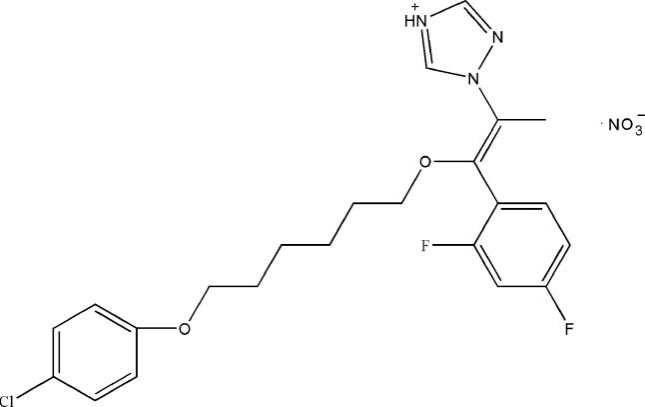


## Experimental

### 

#### Crystal data

C_23_H_25_ClF_2_N_3_O_2_^+^·NO_3_^−^
*M* *_r_* = 510.92
Monoclinic, 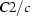

*a* = 35.538 (7) Å
*b* = 8.5550 (17) Å
*c* = 17.072 (3) Å
β = 105.21 (3)°
*V* = 5008.6 (17) Å^3^
*Z* = 8
Mo *K*α radiation
μ = 0.21 mm^−1^
*T* = 293 K
0.30 × 0.20 × 0.10 mm

#### Data collection

Enraf–Nonius CAD-4 diffractometer
Absorption correction: ψ scan (North *et al.*, 1968 ▶) *T*_min_ = 0.940, *T*_max_ = 0.980
9194 measured reflections
4628 independent reflections
1954 reflections with *I* > 2σ(*I*)
*R*_int_ = 0.063
3 standard reflections every 200 reflections  intensity decay: 1%

#### Refinement

*R*[*F* ^2^ > 2σ(*F* ^2^)] = 0.064
*wR*(*F* ^2^) = 0.176
*S* = 1.00
4628 reflections
317 parameters
H-atom parameters constrained
Δρ_max_ = 0.18 e Å^−3^
Δρ_min_ = −0.18 e Å^−3^

### 

Data collection: *CAD-4 EXPRESS* (Enraf–Nonius, 1994 ▶); cell refinement: *CAD-4 EXPRESS*; data reduction: *XCAD4* (Harms & Wocadlo, 1995 ▶); program(s) used to solve structure: *SHELXS97* (Sheldrick, 2008 ▶); program(s) used to refine structure: *SHELXL97* (Sheldrick, 2008 ▶); molecular graphics: *SHELXTL* (Sheldrick, 2008 ▶); software used to prepare material for publication: *PLATON* (Spek, 2009 ▶).

## Supplementary Material

Crystal structure: contains datablock(s) global, I. DOI: 10.1107/S1600536811034933/nc2240sup1.cif

Structure factors: contains datablock(s) I. DOI: 10.1107/S1600536811034933/nc2240Isup2.hkl

Supplementary material file. DOI: 10.1107/S1600536811034933/nc2240Isup3.cml

Additional supplementary materials:  crystallographic information; 3D view; checkCIF report

## Figures and Tables

**Table 1:** Hydrogen-bond geometry (Å, °)

*D*—H⋯*A*	*D*—H	H⋯*A*	*D*⋯*A*	*D*—H⋯*A*
N3—H3*A*⋯O4^i^	0.86	1.8	2.661 (5)	175
C22—H22*A*⋯O4^ii^	0.93	2.38	3.077 (6)	131

Symmetry codes: (i) 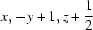; (ii) 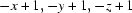.

**Fig. 1.**
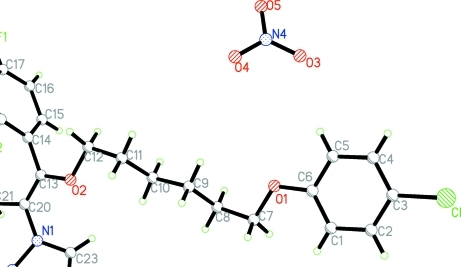
The molecular structure of the title molecule, with the atom numbering scheme. Displacement ellipsoids are drawn at 30% probability levels.

**Fig. 2.**
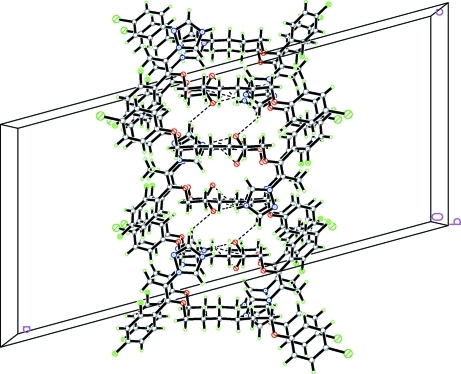
The packing diagram of the title compound. Hydron bonds are shown as dashed lines.

## supplementary crystallographic information

### Comment

Triazole derivatives such as Voriconazole ((*2R,3S*)-2-(2,4-difluorophenyl) -3-(5-fluoropyrimidin-4-yl)-1-(1*H*-1,2,4-triazol-1-yl) butan-2-ol) and Posaconazole (4-(4-(4-(4-(((*3R,5R*)-5-(2,4-difluorophenyl)-5-(1,2,4- triazol-1-ylmethyl)oxolan-3-yl)methoxy)phenyl)piperazin-1-yl)phenyl)- 2-((*2S,3S*)-2-hydroxypentan-3-yl)-1,2,4-triazol-3-one) are safe and effective antifungal agents. (Jeu *et al.*, 2003; Fromtling & Castaner, 1996) As part of our studies on the synthesis of new triazole derivatives, the crystal structure of the title compound was determined.

In the molecular structure of the title compound the double bond is Z configurated. In the crystal structure the anions and cations are connected via N—H···O hydrogen bonding and weak and C—H···O interactions (Table 1 and Fig. 2).

### Experimental

Details on the synthesis can be found in the literature reported by Zirngibl & Thiele (1985). 3 g (0.01 mol) 1-(2,4-difluorophenyl)-2-(1,2,4-triazol)-1-y1)propan-1-one, 10 g of a 50% aqueous sodium hydroxide, 15 ml toluene and 1.5 ml of a 40% aqueous solution of tetrabutyl ammonium hydroxide are mixed and heated to 323.15 K under vigorous stirring. 3.0 g (0.01 mol) 1-bromo-6- (4-chlorophenoxy)-hexane, dissolved in 10 ml toluene, is instilled into the stirred and warmed solution in the course of 10 h. The mixture is subsequently stirred for another 20 h at 323.15 K. The reaction mixture is mixed with as much water and chloroform so that the aqueous phase becomes lighter than the organic phase. Thereafter, the organic and aqueous phases are separated. The organic phase is dried with sodium sulfate. The solvents are distilled under reduced pressure. The remaining residue is a dark oil that is diluted with 10 ml 2-propanol and then adjusted to a PH-value of 2 by means of 30% aqueous nitric acid. The thus derived nitric acid solution is then cooled in the refrigerator. The impure precipitated product herein is subsequently crystallized from a 1:1 mixture of ethyl acetate and ethanol. The purified product may be analytically identified as an approximately pure *Z*-isomer of propylene nitrate. Crystals of title compound suitable for X-ray diffraction were obtained by slow evaporation of an ethanol solution.

### Refinement

H atoms were positioned geometrically with C—H = 0.93 and 0.97 Å for aromatic and methylene H atoms, respectively, and with N—H = 0.86 Å for triazole H atom, and constrained to ride on their parent atoms, with *U*_iso_(H) = 1.2 (or 1.5 for methyl groups) times *U*_eq_(C).

### Crystal data

**Table:**

C_23_H_25_ClF_2_N_3_O_2_^+^·NO_3_^−^	*F*(000) = 2128
*M**_r_* = 510.92	*D*_x_ = 1.355 Mg m^−^^3^
Monoclinic, *C*2/*c*	Melting point: 383.15 K
Hall symbol: -C 2yc	Mo *K*α radiation, λ = 0.71073 Å
*a* = 35.538 (7) Å	Cell parameters from 25 reflections
*b* = 8.5550 (17) Å	θ = 9–13°
*c* = 17.072 (3) Å	µ = 0.21 mm^−^^1^
β = 105.21 (3)°	*T* = 293 K
*V* = 5008.6 (17) Å^3^	Block, yellow
*Z* = 8	0.30 × 0.20 × 0.10 mm

### Data collection

**Table:**

Enraf–Nonius CAD-4 diffractometer	1954 reflections with *I* > 2σ(*I*)
Radiation source: fine-focus sealed tube	*R*_int_ = 0.063
graphite	θ_max_ = 25.5°, θ_min_ = 1.2°
ω/2θ scans	*h* = −42→42
Absorption correction: ψ scan (North *et al.*, 1968)	*k* = −10→0
*T*_min_ = 0.940, *T*_max_ = 0.980	*l* = −20→20
9194 measured reflections	3 standard reflections every 200 reflections
4628 independent reflections	intensity decay: 1%

### Refinement

**Table:**

Refinement on *F*^2^	Secondary atom site location: difference Fourier map
Least-squares matrix: full	Hydrogen site location: inferred from neighbouring sites
*R*[*F*^2^ > 2σ(*F*^2^)] = 0.064	H-atom parameters constrained
*wR*(*F*^2^) = 0.176	*w* = 1/[σ^2^(*F*_o_^2^) + (0.068*P*)^2^] where *P* = (*F*_o_^2^ + 2*F*_c_^2^)/3
*S* = 1.00	(Δ/σ)_max_ < 0.001
4628 reflections	Δρ_max_ = 0.18 e Å^−^^3^
317 parameters	Δρ_min_ = −0.18 e Å^−^^3^
0 restraints	Extinction correction: *SHELXL97* (Sheldrick, 2008), Fc^*^=kFc[1+0.001xFc^2^λ^3^/sin(2θ)]^-1/4^
Primary atom site location: structure-invariant direct methods	Extinction coefficient: 0.0017 (3)

### Special details

**Table:**

Geometry. All e.s.d.'s (except the e.s.d. in the dihedral angle between two l.s. planes) are estimated using the full covariance matrix. The cell e.s.d.'s are taken into account individually in the estimation of e.s.d.'s in distances, angles and torsion angles; correlations between e.s.d.'s in cell parameters are only used when they are defined by crystal symmetry. An approximate (isotropic) treatment of cell e.s.d.'s is used for estimating e.s.d.'s involving l.s. planes.
Refinement. Refinement of *F*^2^ against ALL reflections. The weighted *R*-factor *wR* and goodness of fit *S* are based on *F*^2^, conventional *R*-factors *R* are based on *F*, with *F* set to zero for negative *F*^2^. The threshold expression of *F*^2^ > σ(*F*^2^) is used only for calculating *R*-factors(gt) *etc*. and is not relevant to the choice of reflections for refinement. *R*-factors based on *F*^2^ are statistically about twice as large as those based on *F*, and *R*- factors based on ALL data will be even larger.

### Fractional atomic coordinates and isotropic or equivalent isotropic displacement parameters (Å^2^)

**Table:**

	*x*	*y*	*z*	*U*_iso_*/*U*_eq_	
Cl	0.24234 (4)	0.19455 (19)	0.11286 (9)	0.1265 (6)	
N1	0.60062 (8)	0.8994 (3)	0.62210 (18)	0.0581 (8)	
O1	0.39276 (8)	0.4929 (4)	0.25522 (17)	0.0823 (9)	
F1	0.73827 (8)	0.7269 (3)	0.34649 (16)	0.1146 (10)	
C1	0.32631 (12)	0.4430 (5)	0.2609 (2)	0.0796 (13)	
H1A	0.3293	0.5017	0.3080	0.096*	
O2	0.58960 (7)	0.7156 (3)	0.48863 (16)	0.0806 (9)	
F2	0.68002 (7)	0.5624 (3)	0.54599 (15)	0.1046 (9)	
N2	0.61212 (9)	0.9274 (4)	0.70427 (19)	0.0790 (10)	
C2	0.29134 (12)	0.3703 (6)	0.2254 (3)	0.0834 (13)	
H2B	0.2707	0.3798	0.2493	0.100*	
N3	0.54892 (9)	0.8995 (4)	0.6624 (2)	0.0728 (10)	
H3A	0.5249	0.8934	0.6638	0.087*	
C3	0.28647 (12)	0.2854 (6)	0.1564 (3)	0.0805 (13)	
C4	0.31692 (13)	0.2685 (5)	0.1204 (3)	0.0774 (12)	
H4A	0.3137	0.2095	0.0734	0.093*	
C5	0.35210 (11)	0.3405 (5)	0.1553 (2)	0.0693 (11)	
H5A	0.3727	0.3306	0.1314	0.083*	
C6	0.35694 (11)	0.4270 (5)	0.2254 (2)	0.0666 (11)	
C7	0.39858 (12)	0.5892 (6)	0.3251 (3)	0.0944 (14)	
H7A	0.3968	0.5268	0.3714	0.113*	
H7B	0.3786	0.6692	0.3162	0.113*	
C8	0.43785 (13)	0.6638 (6)	0.3416 (3)	0.1040 (16)	
H8A	0.4391	0.7249	0.2945	0.125*	
H8B	0.4406	0.7357	0.3867	0.125*	
C9	0.47111 (12)	0.5551 (6)	0.3607 (3)	0.0940 (15)	
H9A	0.4698	0.4879	0.3143	0.113*	
H9B	0.4695	0.4895	0.4060	0.113*	
C10	0.51068 (13)	0.6424 (6)	0.3825 (3)	0.1051 (16)	
H10A	0.5125	0.7054	0.3364	0.126*	
H10B	0.5114	0.7126	0.4275	0.126*	
C11	0.54449 (13)	0.5380 (6)	0.4046 (3)	0.1036 (16)	
H11A	0.5423	0.4622	0.3613	0.124*	
H11B	0.5435	0.4810	0.4531	0.124*	
C12	0.58357 (12)	0.6168 (6)	0.4198 (3)	0.1026 (16)	
H12A	0.6040	0.5384	0.4288	0.123*	
H12B	0.5847	0.6779	0.3726	0.123*	
C13	0.62371 (10)	0.7959 (5)	0.5136 (2)	0.0553 (9)	
C14	0.65339 (10)	0.7764 (5)	0.4670 (2)	0.0562 (10)	
C15	0.65451 (12)	0.8748 (5)	0.4041 (2)	0.0796 (12)	
H15A	0.6361	0.9540	0.3894	0.095*	
C16	0.68307 (14)	0.8571 (6)	0.3618 (3)	0.0880 (14)	
H16A	0.6835	0.9218	0.3182	0.106*	
C17	0.71014 (12)	0.7424 (6)	0.3862 (3)	0.0754 (13)	
C18	0.71030 (11)	0.6422 (5)	0.4471 (3)	0.0742 (12)	
H18A	0.7289	0.5638	0.4622	0.089*	
C19	0.68107 (11)	0.6632 (5)	0.4859 (2)	0.0638 (11)	
C20	0.62999 (10)	0.8855 (5)	0.5780 (2)	0.0584 (10)	
C21	0.66594 (11)	0.9797 (6)	0.6133 (3)	0.0979 (16)	
H21A	0.6842	0.9647	0.5813	0.147*	
H21B	0.6775	0.9464	0.6680	0.147*	
H21C	0.6592	1.0883	0.6131	0.147*	
C22	0.57978 (13)	0.9257 (5)	0.7252 (3)	0.0823 (13)	
H22A	0.5781	0.9410	0.7781	0.099*	
C23	0.56259 (11)	0.8848 (5)	0.5979 (2)	0.0700 (11)	
H23A	0.5479	0.8672	0.5450	0.084*	
N4	0.45438 (12)	0.1212 (5)	0.0878 (3)	0.0839 (11)	
O3	0.41881 (10)	0.1263 (5)	0.0716 (2)	0.1251 (13)	
O4	0.47371 (9)	0.1062 (5)	0.1602 (2)	0.1148 (13)	
O5	0.47201 (9)	0.1337 (4)	0.03508 (19)	0.1087 (11)	

### Atomic displacement parameters (Å^2^)

**Table:**

	*U*^11^	*U*^22^	*U*^33^	*U*^12^	*U*^13^	*U*^23^
Cl	0.0756 (9)	0.1377 (13)	0.1570 (13)	−0.0353 (8)	0.0145 (8)	−0.0120 (10)
N1	0.0538 (19)	0.066 (2)	0.058 (2)	0.0018 (16)	0.0214 (15)	−0.0005 (17)
O1	0.0680 (19)	0.100 (2)	0.0800 (19)	−0.0165 (17)	0.0211 (14)	−0.0192 (18)
F1	0.1021 (19)	0.133 (2)	0.142 (2)	−0.0203 (17)	0.0900 (18)	−0.0211 (19)
C1	0.071 (3)	0.106 (4)	0.068 (3)	−0.006 (3)	0.028 (2)	−0.001 (3)
O2	0.0637 (18)	0.101 (2)	0.088 (2)	−0.0172 (16)	0.0399 (15)	−0.0352 (18)
F2	0.0991 (19)	0.124 (2)	0.1046 (19)	0.0447 (16)	0.0508 (15)	0.0433 (17)
N2	0.070 (2)	0.114 (3)	0.060 (2)	0.001 (2)	0.0299 (18)	−0.004 (2)
C2	0.063 (3)	0.106 (4)	0.089 (3)	−0.011 (3)	0.034 (2)	0.014 (3)
N3	0.059 (2)	0.080 (3)	0.091 (3)	−0.0028 (19)	0.040 (2)	−0.006 (2)
C3	0.064 (3)	0.086 (3)	0.091 (3)	−0.014 (2)	0.018 (3)	0.007 (3)
C4	0.079 (3)	0.073 (3)	0.077 (3)	−0.010 (3)	0.013 (2)	−0.003 (2)
C5	0.067 (3)	0.072 (3)	0.074 (3)	−0.005 (2)	0.027 (2)	0.000 (2)
C6	0.054 (2)	0.074 (3)	0.070 (3)	−0.009 (2)	0.013 (2)	0.004 (2)
C7	0.077 (3)	0.105 (4)	0.096 (3)	−0.015 (3)	0.015 (3)	−0.019 (3)
C8	0.084 (4)	0.101 (4)	0.113 (4)	−0.013 (3)	0.001 (3)	−0.024 (3)
C9	0.069 (3)	0.106 (4)	0.099 (3)	−0.020 (3)	0.008 (2)	−0.005 (3)
C10	0.080 (3)	0.113 (4)	0.115 (4)	−0.015 (3)	0.014 (3)	−0.019 (3)
C11	0.077 (3)	0.119 (4)	0.117 (4)	−0.014 (3)	0.029 (3)	−0.023 (3)
C12	0.063 (3)	0.139 (4)	0.109 (4)	−0.017 (3)	0.029 (3)	−0.050 (4)
C13	0.046 (2)	0.063 (3)	0.059 (2)	0.001 (2)	0.0191 (18)	0.002 (2)
C14	0.052 (2)	0.065 (3)	0.057 (2)	0.001 (2)	0.0227 (18)	0.002 (2)
C15	0.079 (3)	0.084 (3)	0.085 (3)	0.012 (2)	0.038 (2)	0.012 (3)
C16	0.103 (4)	0.093 (4)	0.083 (3)	−0.011 (3)	0.052 (3)	0.010 (3)
C17	0.066 (3)	0.084 (4)	0.091 (3)	−0.019 (3)	0.046 (3)	−0.020 (3)
C18	0.053 (2)	0.085 (3)	0.091 (3)	−0.001 (2)	0.031 (2)	−0.011 (3)
C19	0.061 (2)	0.075 (3)	0.059 (2)	0.001 (2)	0.023 (2)	0.006 (2)
C20	0.049 (2)	0.073 (3)	0.058 (2)	0.004 (2)	0.0211 (18)	0.001 (2)
C21	0.067 (3)	0.132 (4)	0.105 (3)	−0.030 (3)	0.040 (2)	−0.040 (3)
C22	0.083 (3)	0.109 (4)	0.064 (3)	−0.008 (3)	0.036 (3)	−0.011 (3)
C23	0.051 (2)	0.094 (3)	0.070 (3)	−0.001 (2)	0.025 (2)	−0.011 (3)
N4	0.071 (3)	0.099 (3)	0.091 (3)	−0.004 (2)	0.037 (2)	−0.015 (3)
O3	0.064 (2)	0.180 (4)	0.134 (3)	−0.024 (2)	0.0314 (19)	−0.032 (3)
O4	0.076 (2)	0.192 (4)	0.089 (2)	0.014 (2)	0.0453 (19)	0.026 (3)
O5	0.092 (2)	0.156 (3)	0.094 (2)	0.004 (2)	0.0514 (19)	0.001 (2)

### Geometric parameters (Å, °)

**Table:**

Cl—C3	1.733 (4)	C9—H9A	0.9700
N1—C23	1.311 (4)	C9—H9B	0.9700
N1—N2	1.375 (4)	C10—C11	1.465 (6)
N1—C20	1.443 (4)	C10—H10A	0.9700
O1—C6	1.363 (4)	C10—H10B	0.9700
O1—C7	1.420 (5)	C11—C12	1.503 (5)
F1—C17	1.353 (4)	C11—H11A	0.9700
C1—C2	1.379 (5)	C11—H11B	0.9700
C1—C6	1.385 (5)	C12—H12A	0.9700
C1—H1A	0.9300	C12—H12B	0.9700
O2—C13	1.361 (4)	C13—C20	1.310 (5)
O2—C12	1.418 (5)	C13—C14	1.488 (5)
F2—C19	1.349 (4)	C14—C19	1.358 (5)
N2—C22	1.290 (4)	C14—C15	1.374 (5)
C2—C3	1.357 (5)	C15—C16	1.400 (5)
C2—H2B	0.9300	C15—H15A	0.9300
N3—C23	1.321 (4)	C16—C17	1.361 (6)
N3—C22	1.335 (5)	C16—H16A	0.9300
N3—H3A	0.8600	C17—C18	1.347 (5)
C3—C4	1.385 (6)	C18—C19	1.381 (5)
C4—C5	1.381 (5)	C18—H18A	0.9300
C4—H4A	0.9300	C20—C21	1.497 (5)
C5—C6	1.378 (5)	C21—H21A	0.9600
C5—H5A	0.9300	C21—H21B	0.9600
C7—C8	1.492 (5)	C21—H21C	0.9600
C7—H7A	0.9700	C22—H22A	0.9300
C7—H7B	0.9700	C23—H23A	0.9300
C8—C9	1.472 (6)	N4—O3	1.222 (4)
C8—H8A	0.9700	N4—O4	1.253 (4)
C8—H8B	0.9700	N4—O5	1.229 (4)
C9—C10	1.548 (6)		
			
C23—N1—N2	110.1 (3)	C10—C11—C12	115.4 (5)
C23—N1—C20	130.9 (3)	C10—C11—H11A	108.4
N2—N1—C20	119.0 (3)	C12—C11—H11A	108.4
C6—O1—C7	118.1 (3)	C10—C11—H11B	108.4
C2—C1—C6	119.0 (4)	C12—C11—H11B	108.4
C2—C1—H1A	120.5	H11A—C11—H11B	107.5
C6—C1—H1A	120.5	O2—C12—C11	110.0 (4)
C13—O2—C12	119.3 (3)	O2—C12—H12A	109.7
C22—N2—N1	103.4 (3)	C11—C12—H12A	109.7
C3—C2—C1	121.2 (4)	O2—C12—H12B	109.7
C3—C2—H2B	119.4	C11—C12—H12B	109.7
C1—C2—H2B	119.4	H12A—C12—H12B	108.2
C23—N3—C22	106.3 (3)	C20—C13—O2	120.0 (3)
C23—N3—H3A	126.9	C20—C13—C14	121.7 (3)
C22—N3—H3A	126.9	O2—C13—C14	118.3 (3)
C2—C3—C4	120.3 (4)	C19—C14—C15	117.0 (3)
C2—C3—Cl	120.5 (4)	C19—C14—C13	121.7 (3)
C4—C3—Cl	119.2 (4)	C15—C14—C13	121.3 (4)
C3—C4—C5	119.1 (4)	C14—C15—C16	120.7 (4)
C3—C4—H4A	120.4	C14—C15—H15A	119.6
C5—C4—H4A	120.4	C16—C15—H15A	119.6
C6—C5—C4	120.5 (4)	C17—C16—C15	118.0 (4)
C6—C5—H5A	119.8	C17—C16—H16A	121.0
C4—C5—H5A	119.8	C15—C16—H16A	121.0
O1—C6—C5	116.0 (3)	C18—C17—F1	118.1 (5)
O1—C6—C1	124.0 (4)	C18—C17—C16	123.7 (4)
C5—C6—C1	120.0 (4)	F1—C17—C16	118.2 (5)
O1—C7—C8	109.3 (4)	C17—C18—C19	115.8 (4)
O1—C7—H7A	109.8	C17—C18—H18A	122.1
C8—C7—H7A	109.8	C19—C18—H18A	122.1
O1—C7—H7B	109.8	F2—C19—C14	118.3 (3)
C8—C7—H7B	109.8	F2—C19—C18	117.1 (4)
H7A—C7—H7B	108.3	C14—C19—C18	124.6 (4)
C9—C8—C7	115.4 (4)	C13—C20—N1	119.9 (3)
C9—C8—H8A	108.4	C13—C20—C21	126.3 (3)
C7—C8—H8A	108.4	N1—C20—C21	113.8 (3)
C9—C8—H8B	108.4	C20—C21—H21A	109.5
C7—C8—H8B	108.4	C20—C21—H21B	109.5
H8A—C8—H8B	107.5	H21A—C21—H21B	109.5
C8—C9—C10	112.0 (4)	C20—C21—H21C	109.5
C8—C9—H9A	109.2	H21A—C21—H21C	109.5
C10—C9—H9A	109.2	H21B—C21—H21C	109.5
C8—C9—H9B	109.2	N2—C22—N3	112.6 (4)
C10—C9—H9B	109.2	N2—C22—H22A	123.7
H9A—C9—H9B	107.9	N3—C22—H22A	123.7
C11—C10—C9	113.6 (4)	N1—C23—N3	107.6 (3)
C11—C10—H10A	108.8	N1—C23—H23A	126.2
C9—C10—H10A	108.8	N3—C23—H23A	126.2
C11—C10—H10B	108.8	O3—N4—O4	119.6 (4)
C9—C10—H10B	108.8	O3—N4—O5	121.8 (4)
H10A—C10—H10B	107.7	O4—N4—O5	118.6 (4)
			
C23—N1—N2—C22	0.9 (5)	C19—C14—C15—C16	0.2 (6)
C20—N1—N2—C22	−177.0 (3)	C13—C14—C15—C16	178.7 (4)
C6—C1—C2—C3	−0.6 (7)	C14—C15—C16—C17	−1.6 (7)
C1—C2—C3—C4	0.7 (7)	C15—C16—C17—C18	2.0 (7)
C1—C2—C3—Cl	179.8 (3)	C15—C16—C17—F1	−178.7 (4)
C2—C3—C4—C5	−0.6 (7)	F1—C17—C18—C19	179.8 (3)
Cl—C3—C4—C5	−179.8 (3)	C16—C17—C18—C19	−0.9 (6)
C3—C4—C5—C6	0.5 (6)	C15—C14—C19—F2	−178.2 (3)
C7—O1—C6—C5	177.0 (4)	C13—C14—C19—F2	3.2 (5)
C7—O1—C6—C1	−2.8 (6)	C15—C14—C19—C18	1.1 (6)
C4—C5—C6—O1	179.8 (3)	C13—C14—C19—C18	−177.5 (4)
C4—C5—C6—C1	−0.4 (6)	C17—C18—C19—F2	178.6 (3)
C2—C1—C6—O1	−179.7 (4)	C17—C18—C19—C14	−0.7 (6)
C2—C1—C6—C5	0.4 (6)	O2—C13—C20—N1	−0.3 (5)
C6—O1—C7—C8	−173.4 (4)	C14—C13—C20—N1	−179.1 (3)
O1—C7—C8—C9	−62.7 (6)	O2—C13—C20—C21	179.5 (4)
C7—C8—C9—C10	−176.3 (4)	C14—C13—C20—C21	0.7 (6)
C8—C9—C10—C11	178.0 (4)	C23—N1—C20—C13	−27.6 (6)
C9—C10—C11—C12	175.4 (4)	N2—N1—C20—C13	149.8 (3)
C13—O2—C12—C11	178.8 (4)	C23—N1—C20—C21	152.6 (4)
C10—C11—C12—O2	64.8 (6)	N2—N1—C20—C21	−30.0 (5)
C12—O2—C13—C20	−178.8 (4)	N1—N2—C22—N3	−0.3 (5)
C12—O2—C13—C14	0.1 (5)	C23—N3—C22—N2	−0.4 (5)
C20—C13—C14—C19	89.2 (5)	N2—N1—C23—N3	−1.2 (5)
O2—C13—C14—C19	−89.7 (4)	C20—N1—C23—N3	176.4 (3)
C20—C13—C14—C15	−89.3 (5)	C22—N3—C23—N1	1.0 (5)
O2—C13—C14—C15	91.8 (5)		

### Hydrogen-bond geometry (Å, °)

**Table:**

*D*—H···*A*	*D*—H	H···*A*	*D*···*A*	*D*—H···*A*
N3—H3A···O4^i^	0.86	1.8	2.661 (5)	175
C22—H22A···O4^ii^	0.93	2.38	3.077 (6)	131

Symmetry codes: (i) *x*, −*y*+1, *z*+1/2; (ii) −*x*+1, −*y*+1, −*z*+1.
